# Japanese translation and validation of web-based questionnaires on overuse injuries and health problems

**DOI:** 10.1371/journal.pone.0242993

**Published:** 2020-12-03

**Authors:** Sonoko Mashimo, Naruto Yoshida, Takaaki Hogan, Ayaka Takegami, Junichi Hirono, Yuya Matsuki, Maya Hagiwara, Yasuharu Nagano

**Affiliations:** 1 Institute for Liberal Arts and Sciences, Osaka Electro-Communication University, Neyagawa, Osaka, Japan; 2 Faculty of Health Care, Teikyo Heisei University, Toshima, Tokyo, Japan; 3 Media Communication Center, Osaka Electro-Communication University, Neyagawa, Osaka, Japan; 4 Graduate School of Comprehensive Human Sciences, University of Tsukuba, Tsukuba, Ibaraki, Japan; 5 School of General Education, Shinshu University, Matsumoto, Nagano, Japan; 6 Center for Educational Development, Kyoto University of Advanced Science, Kameoka, Kyoto, Japan; 7 Faculty of Management and Information Science, Niigata University of Management, Kamo, Niigata, Japan; 8 Department of Sports and Health Science, Japan Women’s College of Physical Education, Setagaya, Tokyo, Japan; Hong Kong Polytechnic University, HONG KONG

## Abstract

This study aimed to translate and culturally adapt the Oslo Sports Trauma Research Center Overuse Injury Questionnaire (OSTRC-O) and the Oslo Sports Trauma Research Center Questionnaire on Health Problems (OSTRC-H) into the Japanese context. The validity and reliability of these translated questionnaires examining overuse injuries and health problems among Japanese university athletes were also examined. The translation was performed following an internationally recognized methodology. A total of 145 athletes were tracked over 10 consecutive weeks and four questions were added in the 10th week to examine the questionnaires’ content validity. Test-retest analysis for reliability was performed 24–72 hours after the 10th week of registration. Internal consistency was determined by calculating Cronbach’s *a* during the cohort study. No major disagreements were found in the translation process. The translated questionnaires had high acceptance and compliance, with an average response rate of over 80% throughout the 10-week cohort study. Most participants reported that the questionnaires were not difficult to complete, there were no items they wanted to change or add, and that the web-based technique worked effectively. Good test-retest reliability and high internal consistency was observed in the translated questionnaires. The translated questionnaires were found to be valid, reliable, and acceptable for medically monitoring Japanese athletes.

## Introduction

The sequence of sports injury prevention research outlined by van Mechelen *et al*. in 1992 is to first describe the extent of the problem in terms of the frequency and severity of injuries [1]. Epidemiological methods of investigating the magnitude of injury have not only been published in consensus statements specific to cricket [2, 3], football [4], and rugby union [5], but also in those from the International Olympic Committee as a method of investigating multisport events [6, 7]. To date, the definition of ‘time-loss’, which is the narrowest of all consensus-recommended definitions, has been used in many injury surveillance studies [8–10]. It is common to broadly classify injury type based on its mechanism; injuries associated with a single, identifiable event are referred to as traumatic, and those that are not associated as such are often referred to as overuse injuries [4]. The approach involved in this definition of time-loss particularly underestimates the full impact of overuse injuries because athletes with overuse injuries are often able to continue training and competing despite persistent injury-associated symptoms and limitations [11, 12].

The Oslo Sports Trauma Research Center Overuse Injury Questionnaire (OSTRC-O) was developed by Clarsen *et al*. in 2013 to record the extent of overuse injuries [13]. The method utilized by this questionnaire was to periodically distribute the questionnaire to all subjects throughout the course of a surveillance, using a broad definition of injury to record all physical complaints in predefined anatomical areas. The questionnaire contains four domains which seek to evaluate the consequences of overuse injuries on athletes. By administering the questionnaire at regular intervals (e.g., weekly), clinicians and researchers could monitor how the consequences of overuse injury vary over time. The Oslo Sports Trauma Research Center Questionnaire on Health Problems (OSTRC-H), which records health problems (injury and illness), was also developed in 2014 [14]. This questionnaire was evaluated the effectiveness by Clarsen *et al*. throughout the preparation of Norwegian high-level athletes for the 2012 London Olympics. The questionnaire has since become part of the daily monitoring system for Olympic and Paralympic athletes and is used in the Olympic and Paralympic programs of several countries [12]. It is now used not only by top level athletes, but by amateurs [15, 16] and youth athletes as well [17].

On the other hand, the four domains of OSTRC-O have been translated by Nagano *et al*. in Japan [18]. However, the translation of the other questions and the adaptation of the questionnaire to Japanese culture has not been adequately considered. Therefore, the aim of this study was to translate and culturally adapt the OSTRC-O and OSTRC-H into the Japanese context. The validity and reliability of the questionnaires for studying overuse injuries and health problems among Japanese basketball, handball, football, gymnastics, and kendo athletes were also examined.

## Materials and methods

### The questionnaire

The OSTRC-O was developed to capture the extent of overuse injuries in sports [13]. It consists of four questions that are repeated for each of these three regions of the body—knee, lower back, and shoulder. The four questions record the level of sports participation, training volume, sports performance, and pain. A severity score is then calculated from 0 to 100 based on these four questions. The OSTRC-H was developed by modifying the OSTRC-O to record all types of injuries and illnesses [14]. This questionnaire also consists of four questions, and if an athlete reports a health problem, then he/she has to provide additional information such as the type of problem and its location or main symptoms. The questionnaires were designed to be distributed electronically to all athletes to monitor their problems on a weekly basis irrespective of their current overuse injuries or health problems.

In our cohort, the questionnaires were set up on the Google form platform and the web links were distributed to the athletes via email.

### Ethical approval

This study was approved by the Ethics Committee of Osaka Electro-Communication University (approval number: 19–002). All participants provided written informed consent before participating in the study. The present study was conducted in accordance with the principles outlined in the Declaration of Helsinki.

### Translation

The English version of the OSTRC questionnaires (OSTRC-O, OSTRC-H) [13, 14] were used for Japanese translation. The four key questions in the OSTRC-O were translated into Japanese by Nagano *et al* [18]. Therefore, the remainder of these questionnaires were translated in this study. The translation process was conducted according to the guidelines presented by Beaton *et al*. [19, 20] and the principles of good practice laid down by the International Society for Pharmacoeconomics and Outcome Research [21]. The translation process was as follows:

1. Translation permission. Co-author YN obtained permission to translate the original English OSTRC questionnaires from the developer [13, 14].
2. Forward translation. Three independent bilingual Japanese residents (T1, T2, and T3), with Japanese as their mother tongue, translated all of the English questionnaires into Japanese. T1 and T2 were aware of the concepts being examined, whereas T3 was not [19]. The project manager (SM) was one of the translators and participated in all steps of translations [21].
3. Reconciliation. A consensus meeting was held with the three translators (T1, T2, and T3) to address discrepancies in the forward translations. A written report documented issues in relation to the translation process [19].
4. Back translation. The reconciled forward-translated versions of the Japanese questionnaires were subsequently translated back to English by two translators (BT1 and BT2) belonging to a specialized translation agency (Editage, Cactus Communications). BT1 and BT2 were as fluent in Japanese as their mother tongue, which is English; they had no medical background but did have postgraduate degrees. BT1 and BT2 were both blinded to the purpose of the questionnaires and had not seen the original English version [19].
5. Back translation review. The Japanese and English translated versions were then compared with the original versions to ensure conceptual equivalence; the remaining discrepancies and ambiguities were resolved among the project manager and the forward translators. As a form of quality control, the Japanese and English versions were sent to the original developer [21]. A written report documented issues in relation to the back translation process [19].
6. Cognitive Debriefing. Fourteen university female basketball players (7 players for OSTRC-O.JP and 7 players for OSTRC-H.JP) completed the questionnaires and were interviewed by the project manager to assess the Japanese translated versions. While completing the questionnaires, participants verified whether there were any items in the questionnaires that were difficult to understand, and during the interviews, they confirmed whether the content of the items and understanding of the concepts were appropriate. This procedure provided a validity check and the possibility to amend potential errors and problems and to minimize future response errors and nonresponse [21].
7. Review of cognitive debriefing results and finalization. The results of the cognitive debriefing were reviewed, and the item wordings were modified, as necessary. The final translated version of each questionnaire was prepared. Any issues relating to the interpretation of the questionnaires were documented in writing [21].
8. Proofreading. The final translated versions were proofread and checked for spelling and grammatical errors; the layout was finalized by the project manager, T2, T3, and several independent research sports scientists (for the Japanese versions, please see S1 and S2 Files) [19].
9. Final report. The final report documenting the translation processes was finalized by the project manager.

### Athletes and recruitment

We approached the team coaches or athletic trainers from one university basketball team, one university handball team, two university football teams, one university gymnastics team, and one university kendo team and asked whether they were interested in participating in the study. After they expressed interest in the study, an introductory meeting was held for each team with the coaches or athletic trainers and the athletes to present the purpose of the study and ask their consent to participate. The inclusion criteria were as follows: age over 18 years, competing at sub-elite or elite level, and ability to speak and understand the Japanese language [22]. Athletes were included regardless of whether they had present or previous injuries.

The questionnaires (OSTRC-O.JP, OSTRC-H.JP) are intended to be selected and used depending on the purpose of the research, and both questionnaires are not used at the same time [13, 14]. Therefore, the participants were randomly divided into two groups; OSTRC-O.JP or OSTRC-H.JP. The participant’s demographics are summarized in Table 1.

**Table 1:** Participants characteristics.

	OSTRC-O.JP (*n* = 72)	OSTRC-H.JP (*n* = 73)
Sex (*n*)	Male 36	Male 35
	Female 36	Female 38
Age (mean±SD)	19.7±1.4	19.8 ±1.2
Years of participation (mean±SD)	11.1 ±2.9	11.0 ±2.9
Weekly training hours (mean±SD)	12.5 ±6.6	12.8 ±6.8
Basketball (*n*)	6	8
Handball (*n*)	15	14
Football (*n*)	20	20
Gymnastics (*n*)	11	10
Kendo (*n*)	20	21

OSTRC-O.JP, Oslo Sports Trauma Research Center Overuse Injury Questionnaire Japanese version; OSTRC-H.JP, Oslo Sports Trauma Research Center Questionnaire on Health Problems Japanese version; SD, standard deviation.

### Content validity and reliability

Content validity was recorded in two steps. First, by determining the frequency of overuse injuries for OSTRC-O.JP and health problems for OSTRC-H.JP over 10 weeks of web-based self-reported registrations. Second, by adding the following four dichotomous (yes/no) questions to the questionnaires at the 10th week of registration [22, 23]: (1) Do you consider the questionnaire to contain relevant questions regarding the sport you are participating in? (2) Was the questionnaire difficult to complete? (3) Would you like to exchange or add any question to the questionnaire? If so, please specify; and (4) Do you think that the web-based technique works well? After each question, the athletes were offered space for any of their own comments.

The participants were asked to complete the web-based questionnaires every Sunday over 10 weeks. If no response had been received from an athlete after 2 days, they received an automatic remainder email.

To examine test-retest reliability, the participants completed the questionnaires once more within 24–72 hours of the 10th week of the survey to assess content validity.

### Statistical analysis

The basic information of the participants were presented in mean and standard deviation. The response rate was presented in percentages and 95% confidence interval (95%CI) for all athletes, irrespectively of the sport. The prevalence and severity score of overuse injuries and health problems were calculated once a week based on the OSTRC methodology [13, 14].

The intraclass correlation coefficient (ICC) of the severity score from the four questions on both questionnaires was calculated from the test-retest measurements to analyse reliability. Internal consistency was determined by calculating Cronbach’s *a* with 0 indicating no internal consistency and 1 corresponding to perfect internal consistency. The statistical analysis was performed using SPSS version 25.0 with the significant level set at *p*<0.05.

## Results

### Translation and adaptation

There were no major problems with the forward translation of the questionnaires. Minor discrepancies included the use of present or past tense, synonymic use of nouns to describe the locations or the symptoms of injury and illness, sentence structure, preposition usage; for example, using ‘その他の違和感 = other discomforts’ instead of ‘その他の症状 = other complaints’ or ‘先週1週間 = last week’ instead of ‘過去1週間 = past week’.

There were some intentional changes made to the words, questions, and options in the original questionnaires. In the original version of OSTRC-H, the word ‘health problem’ was changed to ‘physical and mental problem’ because the Japanese translation of the word implied ‘severe disease’. For the medical personnel question in OSTRC-H, the options were changed to make them applicable to not only Olympians but also athletes of all levels in Japan. The changes were as follows; ‘Olympic team doctor’ to ‘Team doctor’, ‘Olympic team physiotherapist’ to ‘Team physiotherapist’, ‘Other Olympiatoppen doctor’ to ‘Other doctor’, ‘Other Olympiatoppen physiotherapist’ to ‘Other physiotherapist’. The fifth option, ‘No report’ was added for athletes who did not have a personal doctor or physiotherapist. Similarly, the open-ended question that allowed for additional information to the Olympic medical team was modified ‘medical team’ instead of ‘Olympic medical team’.

### Cognitive debriefing

The cognitive debriefing of the seven university female basketball players yielded no changes to the OSTRC-O.JP. Contrastingly, the cognitive debriefing of OSTRC-H.JP prompted the following changes:

**Question 6.** Some body areas were unknown (e.g., groin), so we added the relevant picture of body areas.

**Question 7.** The word ‘swollen glands’ was not well understood. Therefore, we added the example ‘swollen lymph glands and tonsils’.

**Question 11.** The term ‘medical team’ was unclear, and therefore we added the following sentence: ‘Medical team is defined as the doctors and physiotherapists on your team’.

### Content validity

Seventy-two subjects for the OSTRC-O.JP and 73 subjects for the OSTRC-H.JP were followed over 10 weeks. The average response rate to the OSTRC-O.JP during those 10 weeks was 81.3% (95%CI, 78.4–84.1), while the response rate on the 10th week was 88.9%. For OSTRC-H.JP, the average response rate was 82.7% (95%CI, 80.0–85.5) while the response rate on the 10th week was 93.2%.

**Fig 1:**
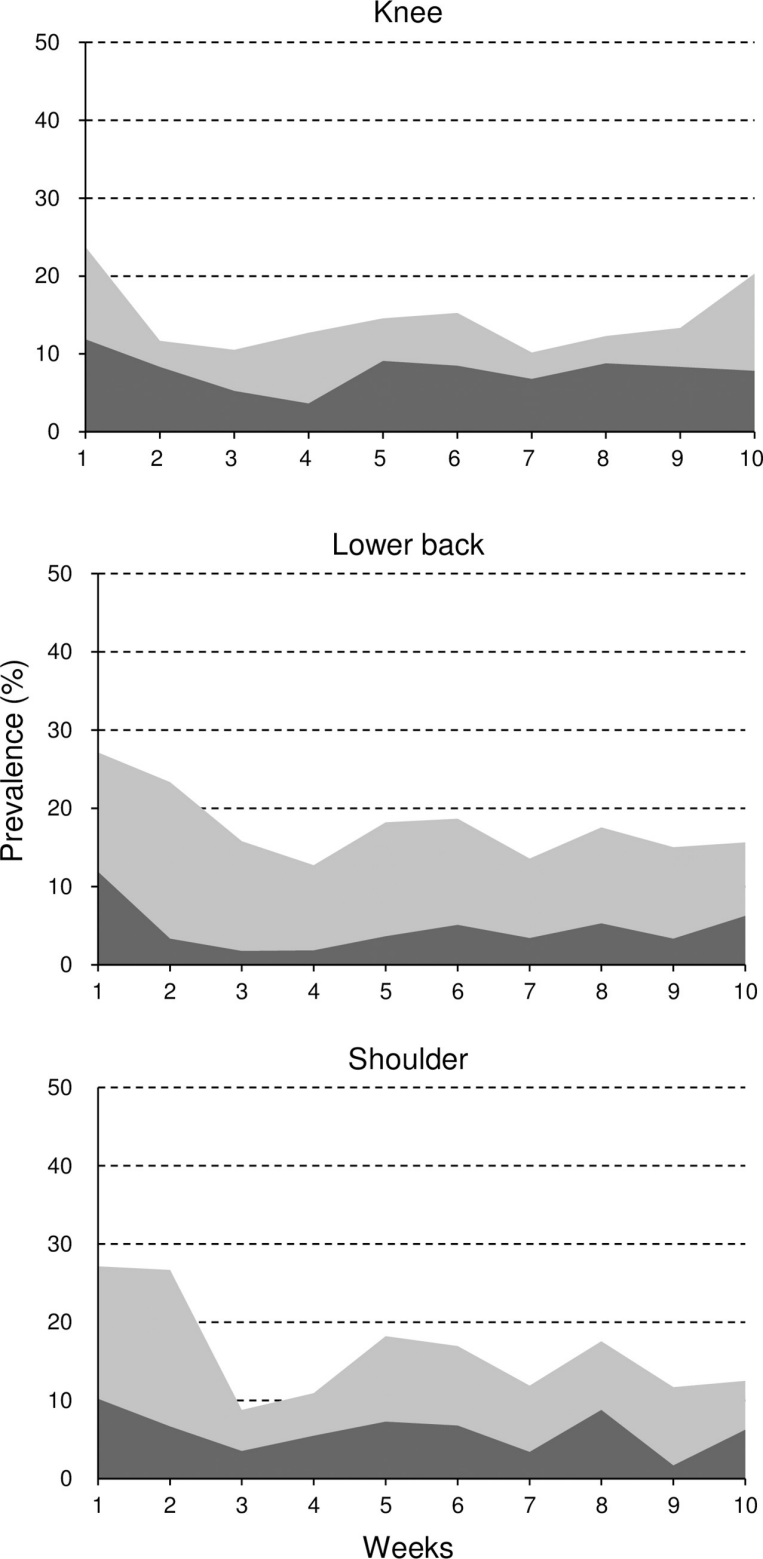
Prevalence of all overuse injuries and substantial overuse injuries over 10 weeks. Light gray area indicated the prevalence of all overuse injuries and dark gray area that of substantial overuse injuries located in the knee, lower back, and shoulder.

**Fig 2:**
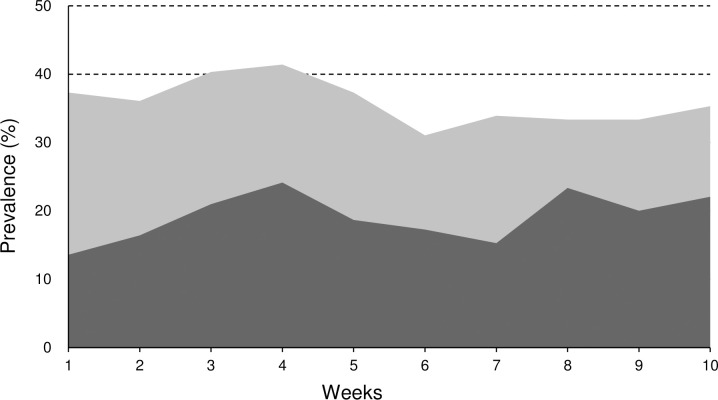
Prevalence of all health problems and substantial health problems over 10 weeks. Light gray area indicated the prevalence of all health problems and dark gray area that of substantial health problems.

The average prevalence of overuse injuries was 14.5% (95%CI, 11.6–17.3) in the knee, 17.8% (95%CI, 14.7–20.8) in the lower back, and 16.2% (95%CI, 13.2–19.2) in the shoulder. The average prevalence of substantial overuse injuries, problems causing moderate/severe reductions in training volume or sports performance, or complete inability to participate in training or competition, was 7.8% (95%CI, 5.7–10.0) in the knee, 4.6% (95%CI, 2.9–6.3) in the lower back, and 6.0% (95%CI, 4.1–7.9) in the shoulder. The average prevalence of health problems was 35.9% (95%CI, 32.1–39.8), and that of substantial health problems was 19.2% (95%CI, 16.0–22.3). The prevalence of overuse injuries by anatomical area and health problems are shown in Figs 1 and 2.

Of the 10th week respondents, 59 subjects in OSTRC-O.JP and 64 subjects in OSTRC-H.JP answered the four additional questions. With exception of four (55/59) on the OSTRC-O.JP and two (62/64) on the OSTRC-H.JP, All the athletes agreed with the questions in the form were relevant to their sports. Almost all participants reported that the questionnaires were not difficult to complete, there were no items they wanted to change or add to the questionnaires, and that the web-based technique worked well. However, some athletes commented that it would be better to develop an application for smart phones. The gymnasts reported that they often have overuse injuries in areas such as the elbow, wrist, and ankle, and wanted to add these areas in the OSTRC-O.JP as they were relevant to their sports.

### Reliability

A test-retest analysis was possible with 30 participants for the OSTRC-O.JP and 41 participants for the OSTRC-H.JP, who completed the questionnaires twice within 24–72 hours. The ICC on the four key questions of the OSTRC-O.JP were 0.89 (95%CI, 0.78–0.95) for the knee, 0.94 (95%CI, 0.88–0.97) for the lower back, and 0.96 (95%CI, 0.92–0.98) for the shoulder. The ICC of OSTRC-H.JP was 0.97 (95%CI, 0.94–0.98).

The internal consistency for the four key questions on the OSTRC-O.JP during the 10 weeks had a mean value of Cronbach’s *a* of 0.97 (95%CI, 0.96–0.98) for the knee, 0.93 (95%CI, 0.91–0.95) for the lower back, and 0.94 (95%CI, 0.93–0.96) for the shoulder. The mean Cronbach’s *a* for the OSTRC-H.JP was 0.96 (95%CI, 0.94–0.98), showing high internal consistency.

## Discussion

The original OSTRC questionnaires were translated and adapted to the Japanese context by using an internationally recognized translation methodology [19–21]. The content validity was examined over a 10-week cohort study and additional questions at 10th week of registration. The average response rates during the cohort period for both questionnaires were very high, which indicated that the Japanese translated questionnaires had high acceptance and compliance. Test-retest reliability was examined during the final week of the cohort study and both questionnaires had high ICCs.

### Translation and adaptation

There were no major disagreements between the original OSTRC and back-translated version of the OSTRC questionnaires, and some deliberate changes were made to adapt them to Japanese sports. There are four key questions in the OSTRC questionnaires that ask about sports participation, training volume, sports performance, and pain. These four key questions in OSTRC-O have already been translated into Japanese [18]. However, other parts of the questionnaires, including the introductions of three body parts in OSTRC-O and the other questions in OSTRC-H had not been translated. Yet these questionnaires have already been translated into Swedish [22], Danish [24], and German [23]. The Japanese translation from the original English questionnaires and the adaptation of them to the Japanese context was carried out using an internationally recognized methodology which was also used in these translation processed [19–21]. A literal translation of ‘health problem’ in Japanese associates it with diseases in our daily lives, such as lifestyle-related diseases. Therefore, the word was changed to ‘physical and mental problem’ to clearly indicate that it includes both physical and mental problems in athletes. We also changed some explanatory texts and options in the questions so that the questionnaires could be used for athletes at various levels, not just Olympic athletes in Japan. A cognitive debriefing was conducted, and the results indicated there was no need for changes in the OSTRC-O.JP. On the other hand, it was pointed out that some words were difficult to understand in the OSTRC-H.JP, and so the figure and additional explanatory texts were added.

No additional questions were added to the questionnaires in the present adaptation process. Contrastingly, in the Swedish translation and adaptation process, two open background questions and three additional questions collecting weekly background data were added. In the German translation process, a question on weekly training volume was added. The original questionnaires have the flexibility to add additional questions according to the clinical and research settings [12–14]. Therefore, it would be desirable to formulate additional questions, if necessary, according to the purpose of the study and its use in the sports field.

### Content validity

Throughout the 10-week cohort study, a high average response rate was observed, which was over 80% for both the OSTRC-O.JP and OSTRC-H.JP. With respect to the high response rate, similar results were obtained from Norwegian, Swedish, and German cohort studies that were conducted over 10 weeks respectively [13, 14, 22, 23, 25]. This suggests that injury and illness web-based registration has high acceptance and compliance.

Content validity was further investigated from the respondent’s perspective through additional four questions at the 10th week of registration [22]. The majority of athletes indicated that the questionnaires included the questions which were relevant to the sport they participate in. They also responded that it was easy to answer the questionnaires, there was no need to add or modify questions, and the web-based technology operated effectively. In this survey, participants received a link to the questionnaires via email every Sunday and non-responding participants received an automated reminder after two days. Such that, even if an athlete forgot to answer, the reminder could prevent he/she from non-responding. Conversely, some participants noted that they would like mobile phone application to be developed instead of receiving the link via email. The gymnasts also commented that they would like the addition of body areas relevant to their sport. With some literature indicating that the addition more body parts in these questionnaires will likely not have a negative impact on content validity [22, 25].

### Reliability

A test-retest analysis was performed on the OSTRC-O.JP and OSTRC-H.JP. No major intraindividual disagreements were observed in the test-retest answers. For both questionnaires, the ICC for the four key questions were mostly above 0.90. The OSTRC questionnaires were designed to report on health problems (injury and illness) and follow their health problems, which are likely to change daily. Jorgensen *et al*. reported lower ICC values after a test-retest period lasting 2 weeks (ICC = 0.62) than a period lasting a week (ICC = 0.72) [24]. A longer period of time may allow too much time for natural changes in the severity of injuries and illnesses to occur, which may make questionnaires seem less reliable than they actually are [23, 24]. On the other hand, a high reliability (ICC = 0.91) was reported when examining a shorter period of time [23]. Several studies have conducted the retest 24 hours after the test in the test-retest analysis [26–28]. Of course, memory effects or a recall bias must be assumed if a shorter period of 1–3 days for retest [23, 29]. However, because intraindividual severity score could vary by one training or match, we decided that it was not appropriate to consider test-retest reliability over a long period of time.

The average Cronbach’s *a* was approximately 0.95 in both questionnaires, indicating a high internal consistency. Similar results were obtained from the original, Danish, and German version of the questionnaires [13, 14, 23, 24].

### Limitations

There are some limitations in this study. First, we were unable to investigate criterion-related validity because there were no other known methods to capture overuse injuries or health problems. This problem has been raised before in the Swedish translation process [22]. Second, the number of subjects reduced when analysing test-retest reliability. It is recommended that the instruments used for individual assessments in clinical practice should have a high test-retest reliability. A sample of 49 respondents would be sufficient to perform statistical calculations, assuming that such a sample would have a power of 0.80, an expected reliability of 0.90, and a significance level of 0.05 [24]. We assumed a certain number of non-respondents for the test-retest reliability in the last week of the ongoing cohort study and included 72 participants for the OSTRC-O.JP and 73 participants for the OSTRC-H.JP. However, in both questionnaires, only half of the subjects were actually eligible for inclusion in the test-retest analysis. This is because we included participants who responded to the questionnaires twice within 24–72 hours to analyse test-retest reliability. The fact that the ICC was high despite the fact that the sample size was insufficient, which in turn reduced statistical power, indicated that the questionnaires had high reliability.

## Conclusion

This study found that the Japanese translated questionnaires were found to be valid, reliable, and acceptable for the use in medical monitoring among Japanese athletes. In the process of translating the original version of the questionnaires into Japanese, it was adapted to Japanese sports and to be used for all level of athletes, not just Olympians. Although the purpose of this study was not to investigate the prevalence of overuse injuries and health problems, it was found that several athletes who were able to compete had overuse injuries and health problems. The questionnaires made it possible to capture the pattern of these problems over time among Japanese athletes. Future studies are needed to use the questionnaires and longitudinally monitor overuse injuries and health problems in a larger population.

## Supporting information

S1 File The Japanese version of a questionnaire for registration of overuse injuries. (PDF)

S2 File The Japanese version of a questionnaire for registration of health problems. (PDF)

S1 Data Data set for OSTRC-O.JP. (XLSX)

S2 Data Data set for OSTRC-H.JP. (XLSX)
