# Supplementary material for: Japanese translation and validation of web-based questionnaires on overuse injuries and health problems
Source: PLoS One. 2020 Dec 3;15(12):e0242993. doi: 10.1371/journal.pone.0242993 (PMC7714361; doi:10.1371/journal.pone.0242993)
Supplement: S1 File — (PDF) [file pone.0242993.s001.pdf]

障害（オーバーユース）に関する質問紙

Part 1 ：膝の問題

あなたの膝に問題があるかどうかに関わらず全ての質問に答えてください。選択肢の中から最適なものを選び、わからない場合でも最もあてはまる答えを選ぶように努めてください。

「膝の問題」とは、片方あるいは両方の膝に関する痛み、うずき、硬さ、腫れ、不安定感/膝崩れ、ロッキング（引っかかり)、またはその他の症状のこととします。

質問 1

過去 1 週間に膝の問題により、通常の練習や試合への参加に影響が出ましたか？

☐ 膝の問題はなく、全ての練習や試合に参加することができた。
☐ 膝の問題はあったが、全ての練習や試合に参加することができた。
☐ 膝の問題があり、練習や試合への参加を減らした。
☐ 膝の問題があり、練習や試合を行うことができなかった。

質問 2

過去 1 週間に膝の問題により、どの程度練習量を減らしましたか？

☐ 全く減らさなかった。
☐ 少し減らした。
☐ 半分程度減らした。
☐ かなり減らした。
☐ 練習や試合が全くできなかった。

質問 3

過去 1 週間に膝の問題が、どの程度パフォーマンスに影響しましたか？

☐ 全く影響しなかった。
☐ 少し影響した。
☐ ある程度影響した。
☐ かなり影響した。
☐ 全く練習や試合ができない程、影響した。

質問 4

過去 1 週間に経験した膝の痛みはどの程度でしたか？

☐ 全く痛みはなかった。
☐ 少し痛みがあった。
☐ 中程度の痛みがあった。
☐ かなりの痛みがあった。

Part 2 ：腰の問題

あなたの腰に問題があるかどうかに関わらず全ての質問に答えてください。選択肢の中から最適なものを選び、わからない場合でも最もあてはまる答えを選ぶように努めてください。
「腰の問題」とは、腰に関する痛み、うずき、硬さ、またはその他の症状のこととします。

質問 1
過去 1 週間に腰の問題により、通常の練習や試合への参加に影響が出ましたか？

☐ 腰の問題はなく、全ての練習や試合に参加することができた。
☐ 腰の問題はあったが、全ての練習や試合に参加することができた。
☐ 腰の問題があり、練習や試合への参加を減らした。
☐ 腰の問題があり、練習や試合を行うことができなかった。

質問 2
過去 1 週間に腰の問題により、どの程度練習量を減らしましたか？

☐ 全く減らさなかった。
☐ 少し減らした。
☐ 半分程度減らした。
☐ かなり減らした。
☐ 練習や試合が全くできなかった。

質問 3
過去 1 週間に腰の問題が、どの程度パフォーマンスに影響しましたか？

☐ 全く影響しなかった。
☐ 少し影響した。
☐ ある程度影響した。
☐ かなり影響した。
☐ 全く練習や試合ができない程、影響した。

質問 4
過去 1 週間に経験した腰の痛みはどの程度でしたか？

☐ 全く痛みはなかった。
☐ 少し痛みがあった。
☐ 中程度の痛みがあった。
☐ かなりの痛みがあった。

Part 3 ：肩の問題

あなたの肩に問題があるかどうかに関わらず全ての質問に答えてください。選択肢の中から最適なものを選び、わからない場合でも最もあてはまる答えを選ぶように努めてください。

「肩の問題」とは、片方あるいは両方の肩に関する痛み、うずき、硬さ、緩さ、またはその他の症状のこととします。

質問 1

過去 1 週間に肩の問題により、通常の練習や試合への参加に影響が出ましたか？

☐ 肩の問題はなく、全ての練習や試合に参加することができた。
☐ 肩の問題はあったが、全ての練習や試合に参加することができた。
☐ 肩の問題があり、練習や試合への参加を減らした。
☐ 肩の問題があり、練習や試合を行うことができなかった。

質問 2

過去 1 週間に肩の問題により、どの程度練習量を減らしましたか？

☐ 全く減らさなかった。
☐ 少し減らした。
☐ 半分程度減らした。
☐ かなり減らした。
☐ 練習や試合が全くできなかった。

質問 3

過去 1 週間に肩の問題が、どの程度パフォーマンスに影響しましたか？

☐ 全く影響しなかった。
☐ 少し影響した。
☐ ある程度影響した。
☐ かなり影響した。
☐ 全く練習や試合ができない程、影響した。

質問 4

過去 1 週間に経験した肩の痛みはどの程度でしたか？

☐ 全く痛みはなかった。
☐ 少し痛みがあった。
☐ 中程度の痛みがあった。
☐ かなりの痛みがあった。
