# Supplementary material for: Japanese translation and validation of web-based questionnaires on overuse injuries and health problems
Source: PLoS One. 2020 Dec 3;15(12):e0242993. doi: 10.1371/journal.pone.0242993 (PMC7714361; doi:10.1371/journal.pone.0242993)
Supplement: S2 File — (PDF) [file pone.0242993.s002.pdf]

身体上の問題に関する質問紙

過去１週間で身体上の問題があったかどうかに関わらず全ての質問に答えてください。選択肢の中から最適なものを選び、わからない場合でも最もあてはまる答えを選ぶように努めてください。
もし複数の疾病あるいは傷害がある場合には、過去１週間で最も悪かった問題について答えてください。その他の問題については質問紙の最後に回答する機会があります。

質問１
過去１週間に傷害、疾病、あるいはその他の身体上の問題により、通常の練習や試合への参加に影響が出ましたか？

☐ 身体上の問題はなく、全ての練習や試合に参加することができた。
☐ 傷害/疾病はあったが、全ての練習や試合に参加することができた。
☐ 傷害/疾病があり、練習や試合への参加を減らした。
☐ 傷害/疾病があり、練習や試合を行うことができなかった。

質問２
過去１週間に傷害、疾病、あるいはその他の身体上の問題により、どの程度練習量を減らしましたか？

☐ 全く減らさなかった。
☐ 少し減らした。
☐ 半分程度減らした。
☐ かなり減らした。
☐ 練習や試合が全くできなかった。

質問３
過去１週間に傷害、疾病、あるいはその他の身体上の問題が、どの程度パフォーマンスに影響しましたか？

☐ 全く影響しなかった。
☐ 少し影響した。
☐ ある程度影響した。
☐ かなり影響した。
☐ 全く練習や試合ができない程、影響した。

質問４
過去１ 週間に経験した身体上の問題の症状はどの程度でしたか？

☐ 全く症状はなかった。
☐ 少し症状があった。
☐ 中程度の症状があった。
☐ かなりの症状があった。

質問５
上記の４つの質問で答えた身体上の問題は傷害または疾病のどちらですか？

□傷害
□疾病

質問６－傷害の部位
あなたの傷害の部位を選んでください。もし傷害がいくつかの部位におよぶ場合は主要な部位を選んでください。もし傷害が複数ある場合は質問紙を分けて別々に回答してください。

☐ 頭部/顔
☐ 頚部
☐ 肩（鎖骨を含む）
☐ 上腕
☐ 肘
☐ 前腕
☐ 手首
☐ 手/指
☐ 胸部/肋骨
☐ 腹部
☐ 胸椎
☐ 腰椎
☐ 骨盤/臀部
☐ 股関節/鼠径部
☐ 大腿
☐ 膝
☐ 下腿
☐ 足首
☐ 足部/つま先
☐ その他

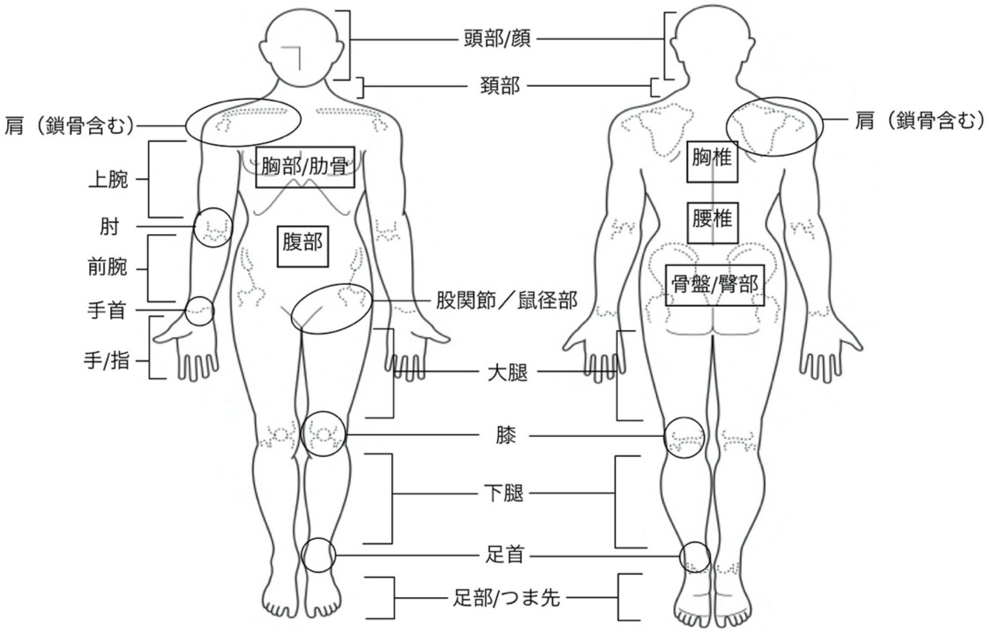

質問７－疾病の症状
過去１週間に経験した主要な疾病の症状を選んでください。複数の選択肢を選ぶことができますが、互いに関係のない疾病が複数ある場合は質問紙を分けて別々に回答してください。

☐ 熱
☐ 疲労/だるさ
☐ 腺の腫れ（リンパ腺や扁桃腺の腫れ等）
☐ のどの痛み
☐ 鼻づまり/鼻水/くしゃみ
☐ 咳
☐ 呼吸困難/息苦しさ

☐ 頭痛
☐ 吐き気
☐ 嘔吐
☐ 下痢
☐ 便秘
☐ 失神
☐ 発疹/かゆみ
☐ 不整脈
☐ 胸痛/狭心症
☐ 腹痛
☐ その他の痛み
☐ 感覚麻痺/しびれ
☐ 不安
☐ 抑うつ/悲しみ
☐ いらつき
☐ 目の症状
☐ 耳の症状
☐ 泌尿器/生殖器の症状
☐ その他。詳しく記入してください＿＿＿＿＿＿＿＿＿＿＿＿＿＿＿＿＿＿＿＿＿＿＿＿＿＿＿

質問 8－不参加日数
過去 1 週間にこの身体上の問題により、練習または試合を完全に休んだ日数を選んでください。

☐ 0　☐ 1　☐ 2　☐ 3　☐ 4　☐ 5　☐ 6　☐ 7

質問 9－報告
この調査システムを通して、この身体上の問題を回答するのは初めてですか？

☐ はい、初めてです。
☐ いいえ、過去 4 週間以内に同じ問題を報告しています。
☐ いいえ、同じ問題を報告していますが、過去 4 週間より前です。

質問 1 0—メディカルスタッフとのコンタクト
この問題について誰に報告しましたか。

☐ 所属チームの医師
☐ 所属チームのトレーナー
☐ その他の医師。名前と所属を記入してください。
☐ その他のトレーナー。名前と所属を記入してください。
☐ 報告していない。

質問１１
この問題についてメディカルチームに知らせたい追加の情報があれば記入してください。（メディカルチームとは，所属チームの医師やトレーナーのこととします）

質問１２
過去１週間にその他の疾病、傷害、あるいは身体上の問題を経験しましたか？

□　はい
□　いいえ
